# Percutaneous Tricuspid Valve Vegetation Debulking in Infective Endocarditis: 2016 to 2020 National Database Analysis

**DOI:** 10.1016/j.jacadv.2023.100373

**Published:** 2023-06-07

**Authors:** Aaqib H. Malik, Dhrubajyoti Bandyopadhyay, Suraj Krishnan, Akshay Goel, Adrija Hajra, Rahul Gupta, Joshua Goldberg, Syed (Abbas) Haidry, Hasan Ahmad

Right-sided endocarditis affects 10% of all cases of infective endocarditis (IE).^1^ About 90% involve the tricuspid valve (TV), and 90% of those are intravenous drug users (IVDUs).^1^ Right-sided IE in IVDUs is increasing, especially in those aged <35 years with higher mortality.^2^ Although medical therapy remains the mainstay, it is considered inadequate in managing large vegetations (>1 cm), persistent bacteremia despite antibiotic therapy, recurrent septic embolization, or fungal lesions.^1^ Percutaneous vegetation debulking may be of novel use in the above subgroup of patients where surgery is indicated; however, not feasible due to perioperative risk and recurrence of events. The Food and Drug Administration approved the AngioVac aspiration system in 2014 for the removal of intravascular material.^3^ Despite the increased utilization of this device in recent years, there are limited data on its outcomes at a national level.

National Inpatient Sample and National Readmission Database from 2016 to 2020 were used with diagnosis codes of IE (International Classification of Diseases, Tenth Revision, Clinical Modification I33x, I38x, and I39x) and procedure code of 02CJ3ZZ to identify hospitalizations for TV-associated debulking. The primary outcome was in-hospital mortality. Secondary outcomes included freedom from TV surgery in index hospitalization, acute kidney injury, cardiac arrest, cardiogenic shock, and 30-day readmissions.

Among 370,790 IE hospitalizations, a total of 1,085 underwent percutaneous debulking of TV. The median age of patients who underwent debulking of the TV was 34.0 years (IQR: 29.0-43.0 years). Most patients were female (57.6%), Caucasians (83%), 3.7% had prior pacemaker or defibrillator, and 38.7% had prior valvular diseases (Table 1). The median length of hospital stay was 20.0 days (IQR: 12.0-39.0 days), while the median cost of hospitalization was $53,062 USD (IQR: $35,367-$87,390 USD).

**Table 1:** Baseline Characteristics and Outcomes

	Infective Endocarditis Without TV Debulking	Infective Endocarditis With TV Debulking	*P* Value
Total population	369,705	1,085	<0.001
Median age, y	61 (42-74)	34 (29-43)	<0.001
Female	43.4	57.6	<0.001
Caucasian	71.4	82.9	<0.001
African American	12.6	6.0	<0.001
Hypertension	60.6	25.3	<0.001
Diabetes	30.0	9.7	<0.001
Obesity	13.5	7.8	0.07
Congestive heart failure	42.4	24.4	<0.001
Valvular heart disease	56.4	38.7	<0.001
Chronic kidney disease	30.5	6.5	<0.001
PVD	10.6	2.3	<0.001
Liver disease	15.4	23.0	<0.001
Hypothyroidism	11.2	2.8	<0.001
Atrial fibrillation	25.3	5.1	<0.001
Prior CVA	10.8	2.3	<0.001
Prior MI	5.8	1.8	<0.05
Prior pacemaker	5.0	2.3	0.11
Prior ICD	2.8	1.4	0.21
Alcohol abuse	4.6	3.7	0.49
Smoking	26.2	51.6	<0.001
Drug abuse	25.4	69.6	<0.001
Outcomes			
Mortality	9.8	4.6	<0.05
PCI	0.6	0.5	0.86
Stroke	3.6	0	0.07
Cardiogenic shock	3.6	5.1	0.25
Cardiac arrest	0.8	0.9	0.81
Ventricular tachycardia	4.6	5.5	0.47
AKI	36.3	46.5	<0.001
AKI on dialysis	4.4	7.4	<0.05
Ventilation more than 24 h	11.7	19.8	<0.001
IABP	0.7	0.5	0.68
ECMO utilization	0.2	13.0	<0.001
Impella	0.1	0	0.69
Pulmonary embolism	13.1	71.9	<0.001
30-d readmission	17.5	17.9	0.77

Values are n or %.

AKI = acute kidney injury; CVA = cerebrovascular accident; IABP = intra-aortic balloon pump; ICD = implantable cardioverter-defibrillator; MI = myocardial infarction; PCI = percutaneous coronary intervention; PVD = peripheral vascular disease; TV = tricuspid valve.

From 2016 to 2020, there was a significant increase in the number of TV debulking procedures from 30 procedures in 2016 to 495 procedures in 2020 (*P* < 0.001). The majority of the procedures (95.4%) were performed in teaching hospitals. In these patients, the in-hospital mortality was 4.6%, and the success rate of the procedure defined as freedom from any TV surgery during index admission was 91%. Among other secondary outcomes, 46.1% had acute kidney injury, 1% had cardiac arrest, 5.1% had a cardiogenic shock, 5.5% had ventricular tachycardia, and 13% required ECMO. The 30-day readmission rate was 18%. The major causes of readmission were infection, sepsis, and endocarditis, accounting for >10% of cases.

In the first nationwide analysis of the TV debulking procedure, there was an exponential increase in this procedure over the last 5 years. We have observed that right-sided TV debulking is a viable option with nearly 90% freedom from in-hospital TV surgery for right-sided IE. This presents this procedure as a viable treatment alternative, especially in IVDUs with recurrent IE and those in whom TV surgery may be prohibitive. As expected, this procedure was primarily utilized in younger patients. Approximately 4.6% of patients died during initial hospitalization, with increased utilization of extracorporeal membrane oxygenation suggesting the selective use of this procedure in higher-risk patients. Approximately 1 in 5 of these patients get readmitted within 30 days, with sepsis and endocarditis accounting for more than 10% of cases.

In IE patients involving TV not amenable to medical therapy, TV repair or valvulotomy is preferred over TV replacement, especially in IVDU, since it reduces the risk of reinfection.^1^ But poor surgical outcomes remain a significant problem due to the recurrence of events. In critically ill patients with higher perioperative risk, percutaneous debulking of TV vegetation may be used.^4^ Also, in patients with device lead-associated TV endocarditis with large vegetation,^3^ TV debulking may be a viable option before lead removal. This novel technique has also been reported as a bridge to cardiac surgery in select cases to reduce the lag time to surgery.^3^ There are limited data on the complications associated with AngioVac device. Using the Manufacturer And User facility Device Experience (MAUDE) database, Dandu et al^5^ recently reported pulmonary embolism and vascular complications to be the most important adverse events associated with the use of this device.

The major limitation of our study holds true for all large administrative database analyses, including coding errors and lack of clinical information, most specifically regarding vegetation size from echocardiography before and after percutaneous TV debulking, rates of pulmonary embolism, or development of pulmonary abscess after the procedure. As with any such nationally representative analysis, the difference in population, institutional practices, or the procedure itself most certainly lack a standardized approach; therefore, any conclusions drawn from this should be cautious. The definition of success of the procedure as freedom from TV surgery is arbitrary and may have been influenced by the severity of the patient’s illness leading to medical management. However, the larger sample size overcomes biases associated with small case reports and series, and historically, procedural data are known to be coded appropriately in these studies.

In this first nationwide analysis, we witnessed increased utilization of TV debulking with approximately 90% freedom from inpatient TV surgery. However, further studies with long-term follow-up are needed.
